# The effects of probiotics on depressive symptoms in humans: a systematic review

**DOI:** 10.1186/s12991-017-0138-2

**Published:** 2017-02-20

**Authors:** Caroline J. K. Wallace, Roumen Milev

**Affiliations:** Department of Psychiatry, Queen’s University, 752 King Street West, Kingston, ON K7L 4X3 Canada

**Keywords:** Depression, Anxiety, Probiotics, Gut–brain axis, Microbiome, Systematic review

## Abstract

**Background:**

Patients suffering from depression experience significant mood, anxiety, and cognitive symptoms. Currently, most antidepressants work by altering neurotransmitter activity in the brain to improve these symptoms. However, in the last decade, research has revealed an extensive bidirectional communication network between the gastrointestinal tract and the central nervous system, referred to as the “gut–brain axis.” Advances in this field have linked psychiatric disorders to changes in the microbiome, making it a potential target for novel antidepressant treatments. The aim of this review is to analyze the current body of research assessing the effects of probiotics, on symptoms of depression in humans.

**Methods:**

A systematic search of five databases was performed and study selection was completed using the preferred reporting items for systematic reviews and meta-analyses process.

**Results:**

Ten studies met criteria and were analyzed for effects on mood, anxiety, and cognition. Five studies assessed mood symptoms, seven studies assessed anxiety symptoms, and three studies assessed cognition. The majority of the studies found positive results on all measures of depressive symptoms; however, the strain of probiotic, the dosing, and duration of treatment varied widely and no studies assessed sleep.

**Conclusion:**

The evidence for probiotics alleviating depressive symptoms is compelling but additional double-blind randomized control trials in clinical populations are warranted to further assess efficacy.

## Background

Major depressive disorder (MDD) is a complex psychiatric disorder of unknown etiology that will affect up to 20% of the population at some point in their lifetime [1], and is a leading cause of disability worldwide [2]. Characterized by low mood or loss of interest and often accompanied by feelings of guilt, hopelessness, and changes in appetite and sleep, MDD significantly impairs daily functioning—including work and school performance and social relationships. Depressive symptoms can also be present subclinically, and still have a major impact on daily functioning. Currently, most pharmacological treatments for MDD focus on altering neurotransmitter activity in the brain, but have a delayed onset of action and can produce adverse side effects such as headaches, nausea, agitation, sedation, and sexual dysfunction [3]. However, in the last decade, neurogastroenterology research has revealed extensive and direct biochemical signaling between the gastrointestinal (GI) tract and the central nervous system, referred to as the “gut–brain axis.” This communication network is bidirectional and occurs via the autonomic nervous system, the enteric nervous system, the neuroendocrine system, and the immune system [4]. These advances have linked central nervous system psychiatric disorders such as MDD to changes in the gastrointestinal microbiome [5–7], making it a potential target for novel antidepressant treatments. This is further corroborated by the high rate of comorbidity between psychiatric disorders and GI disorders [8].

The microbiome, a complex microbial ecosystem containing some 100 trillion microorganisms, functions to establish the intestinal lining and aids in its maintenance [9]. It is influenced by several factors, including genetics [10], age [11], sex [12], diet [13], and, of particular interest lately, stress [14]. There is already evidence that psychological stress can increase permeability of the gastrointestinal lining [15], and conversely, mounting evidence that the microbiome can influence and modulate emotional behavior [16]. As a stress- and emotion-related disorder, this association has led depression researchers to explore the potential of manipulating the microbiome of the GI tract to alleviate depressive symptoms.

Nutritional psychiatry is an emerging field of psychiatry that explores the relationship between dietary patterns and risk of mental health disorders. The brain’s structure and function is dependent on nutrient intake—including amino acids, fats, vitamins, minerals [17], thus diet has emerged as a compelling candidate in regulating mental health [18]. Several cross-sectional studies have used an overall diet approach to evaluate the association between nutrition and mental health [19, 20] but there is also considerable research looking at isolated nutrients and their impact on mental health. Central to this research are probiotics, defined as live microorganisms that, when ingested in adequate amounts, exert a health benefit on the host [21]. Probiotics are transient entities that colonize the GI tract and influence various pathways and are available as a supplement in pill or powder form. It has been well established that probiotics have therapeutic effects on many GI disorders [22]; however, with the emergence of the gut–brain axis, it has been discovered that their therapeutic effects extend beyond the gut and into the central nervous system [23]. There is robust evidence of this in preclinical studies that have demonstrated probiotics’ ability to change behavior and improve the mood, anxiety, and cognition of rodents by altering neurotransmitter activity, reviewed below.

### Preclinical studies

In terms of neurotransmission, several rodent studies found that consumption of probiotics prevented stress-induced increases in adrenocorticotropic hormone (ACTH) [24], corticosterone [25, 26], adrenaline, and noradrenaline [25]. The reduction in these markers of chronic stress suggests that treatment with probiotics attenuated the hypothalamic–pituitary–adrenal (HPA) axis, which is hyperactive in depressed patients [27]. Consumption of probiotics was also shown to increase expression of brain-derived neurotrophic factor (BDNF) [25], a growth factor crucial for brain plasticity, memory, and neuronal health [28] that is abnormally reduced in patients suffering from depression [29]. Other preclinical studies have noted changes in the molecules involved in the biosynthesis and metabolism of the critical neurotransmitter serotonin. Desbonnet and colleagues found that consumption of probiotics increased plasma levels of serotonin’s precursor, tryptophan [30], and, along with Nishino’s group [31], decreased serotonin’s main metabolite 5-hydroxyindoleacetic acid (5-HIAA), similar to the antidepressant citalopram. These findings suggest that probiotics have a positive impact on the central nervous system by regulating critical neurotransmitters implicated in depression. And the central nervous system is not the only physiological system implicated in depression that probiotics have been shown to influence in rodents: consumption of probiotics reduced levels of the pro-inflammatory cytokines interleukin-1-beta (IL-1β) [24, 32] and interleukin-6 (IL-6), as well as tumor necrosis factor-alpha (TNFα) [24] and microglial activation markers [25], indicating they may possess the ability to reduce overall inflammation. Finally, one of the most salient findings in preclinical studies of rodents treated with probiotics are the behavioral and psychological changes they induce, such as improved memory [33, 34] and reduced anxiety- and depressive-like behaviors [26, 32, 35, 36].

Probiotics have also been shown to possess both antioxidant and free radical scavenging abilities [37], increase production of gamma-aminobutyric acid (GABA) [38], and improve absorption of other nutrients [39], all of which have been implicated in the pathophysiology of depression. Moreover, in Western societies, depression is more common among low socioeconomic status populations, who have also been shown to consume less probiotics and fermented foods than their higher SES counterparts, who also experience less depression [40].

These results, along with preclinical and clinical study findings, suggest that gut microbiota and the use of probiotics could prove useful in alleviating depressive symptoms. The objective of this review is to analyze the current body of research assessing the effects of probiotics on symptoms of depression in humans such as mood, anxiety, and cognition, and to identify any gaps and discuss future directions in this field.

## Methods

### Study selection criteria

Articles eligible for inclusion in this review were written or available in English and published in peer-reviewed journals. Conference publications, book chapters, letters, and reviews were excluded. The studies included in the review were restricted to human trials that assessed subjective changes in symptoms of depression such as mood, anxiety, and/or cognition after consuming either a probiotic supplement or a food or drink containing probiotic cultures for an extended period of time. Studies were excluded if investigating GI dysfunctions.

### Search strategy

**Fig. 1:**
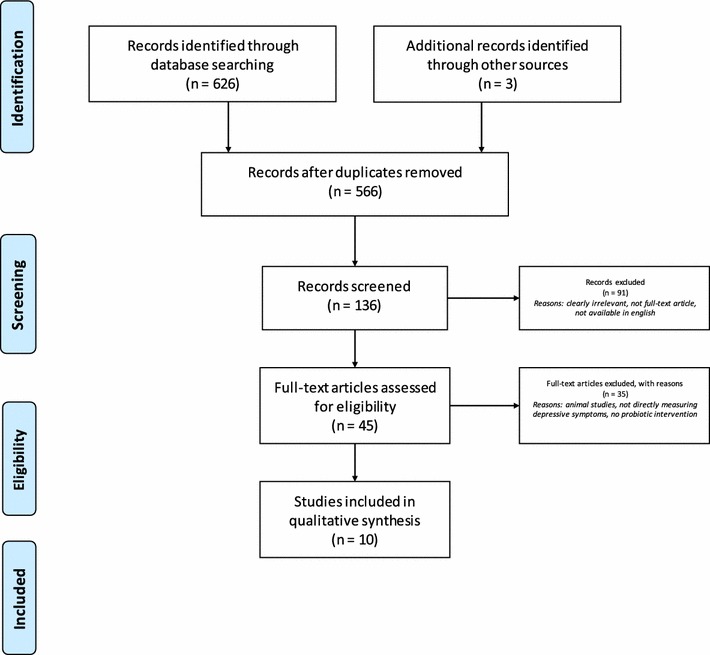
Flow chart of systematic literature search and selection process using preferred reporting items for systematic reviews and meta-analyses (PRISMA) process

A systematic computerized search was performed using the following databases: Medline, PsycINFO, Embase, cumulative index to nursing and allied health literature (CINAHL), and web of science (WoS). A supplementary search of Google Scholar was also performed to ensure no studies were omitted. Key words included depression, mood disorder, emotions, probiotics, *Lactobacillus*, *Bifidobacterium*, and gut–brain axis. The results of the search process are described below (Fig. 1).

### Selection of studies

Study selection was completed using the PRISMA (preferred reporting items for systematic reviews and meta-analyses) process (Fig. 1). One author (C.W.) completed initial screening to exclude irrelevant titles, and remaining abstracts and subsequent remaining full-text articles were independently screened by both authors (C.W. and R. M.) for eligibility in the review. Any disagreement was resolved by discussion between the two authors.

### Quality control of selected studies

Quality of included clinical trials was assessed using the Jadad Scale [41], a widely used scoring system that independently assesses the methodological quality of trials on three measures: randomization, blinding, and withdrawals/dropouts. Trials are scored on a five-point scale and for the purpose of quality control for this review, articles with a score of three or higher were included. All included articles met these criteria. Scores ranged from 3 to 5 with a mean score of 4.1 (*n* = 7). Three additional studies that did not follow a double-blind randomized control trial design were also included due to their sound methodology and analysis of the outcomes as well as to provide the most comprehensive review of the literature.

## Results

This review includes ten studies from five databases listed above. Characteristics of each study are shown in Table 1. The most frequently used probiotic strain was *Lactobacillus casei*, and duration of treatment period ranged from 3 weeks to 6 months. One study assessed patients with depression, two studies assessed adults suffering from stress or exhaustion and chronic fatigue syndrome (CFS), respectively, and the remaining seven studies assessed healthy controls.

**Table 1:** Characteristics of included studies

Reference	Sample characteristics	Strain	Study design	Duration of intervention	Measurement	Key findings and conclusions
Akkasheh et al. [34]	40 MDD patients. Ages 20–55 years	*Lactobacillus acidophilus, L. casei, and Bifidobacterium bifidum*	Double-blind, randomized, placebo-controlled trial	8 weeks	BDI	Consumption of probiotic supplement improved BDI scores
Benton et al. [30]	124 healthy humans. Avg. age: 62 years	*L. casei*	Double-blind, randomized, placebo-controlled trial	3 weeks	POMS, self-rated mood	No effect of probiotic on POMS results. Consumption of probiotic-containing yogurt improved self-reported mood of those whose mood was initially poor
Chung et al. [32]	36 healthy humans. Ages 60–75 years	*L. helveticus*	Double-blind, randomized, placebo-controlled trial	12 weeks	PSS, GDS-SF, DST, SRT, VLT, RVIP, Stroop Task	No significant effects of probiotics on the PSS, GDS-SF. Consumption of probiotics did improve DST, SRT, VLT, RVIP, and stroop tasks scores
Gruenwald et al. [36]	34 adults suffering from stress or exhaustion. Mean age: 44 years	*L. acidophilus* and *B. bifidum* and *longum*	Pre- and post-intervention assessment	6 months	PNQ, EWL	Subjects’ general condition improved by 40.7%. 73% of participants rated the effect of treatment as “good” or “very good”
Hilimire et al. [38]	710 young adults. Mean age: 19 years	Unknown	Self-report questionnaires on fermented food consumption, neuroticism and social anxiety	N/A	BFI, SPAI-23	Consumption of fermented foods containing probiotics was negatively associated with symptoms of social anxiety and interacts with neuroticism to predict social anxiety symptoms. Those at higher genetic risk for social anxiety disorder (indexed by high neuroticism) show fewer social anxiety symptoms when they consume more fermented foods
Marcos et al. [37]	136 healthy students. Ages 18–23 years	*L. casei*	Prospective, randomized, controlled, parallel study	6 weeks	STAI	No significant effects of probiotics on anxiety levels. Probiotics did modulate

*MDD* major depressive disorder, *BDI* Beck Depression Inventory, *POMS* profile of mood states scale, *PSS* perceived stress scale, *GDS-SF* geriatric depression scale, *DST* digit span test, *SRT* story recall test, *VLT* verbal learning test, *RVIP* rapid visual information-processing, *PNQ* psychological-neurologic questionnaire, *EWL* list of adjectives, *bfi* big five inventory, *SPAI-23* social phobia and anxiety inventory, *STAI* state-trait anxiety inventory, *HADS* Hospital Anxiety Depression Scale, *HSCL-90* Hopkins symptom checklist, *CCL* coping checklist, *UFC* urinary free cortisol, *BAI* Beck Anxiety Inventory, *LEIDS-r* Leiden index of depression sensitivity, *fMRI* functional magnetic resonance imaging

### Effects on mood

Of the ten included studies, five assessed mood symptoms and all but two reported improvements after treatment with a probiotic. Benton and colleagues’ 2007 double-blind randomized control trial with 124 healthy humans found that after only 20 days of consuming a probiotic-containing milk drink, although having no effect on the profile of mood states (POMS) scale, participants whose self-rated mood was initially poor had improved [42]. Following Benton, Rao and colleagues conducted a study with 35 patients suffering from CFS who were randomly assigned to receive a probiotic or placebo three times daily for 2 months. Depressive and anxiety symptoms were assessed with the Beck Depression Inventory (BDI) and the Beck Anxiety Inventory (BAI) pre- and post-intervention, and results showed that while consumption of the probiotic improved anxiety scores, it had no effect on depressive symptoms [43]. Similarly, Young-Chul Chung and colleagues implemented a 12-week, double-blind randomized controlled experiment with healthy 60–75 year olds and found no significant improvement in geriatric depression scale (GDS-SF) scores after treatment with a probiotic fermented milk [44]. However, Messaoudi and colleagues’ 2011 research on healthy humans found that consumption of a probiotic supplement reduced somatisation, depression, and anger-hostility scores on the Hopkins symptom checklist (HSCL-90), as well as reduced Hospital Anxiety and Depression Scale (HADS) global scores [36]. Steenbergen and colleagues then went on to conduct a triple-blind (blind at three levels: group allocator, participants, and outcome assessor) randomized control trial assessing the effects of probiotics on cognitive reactivity to sad mood using the Leiden index of depression sensitivity (LEIDS-r). Forty healthy young adults consumed either a probiotic supplement or placebo for 4 weeks and found that consumption of the multispecies probiotic formula significantly reduced overall cognitive reactivity to depression, in particular aggressive and ruminative thoughts [45]. Finally, and most recently, Akkasheh and colleagues [46] designed a double-blind randomized placebo-controlled trial with 40 patients diagnosed with depression. Participants consumed either a probiotic supplement or placebo for 8 weeks and were assessed using the BDI at baseline and post-treatment. Results showed that consumption of the probiotic supplement significantly decreased BDI scores indicating overall improved symptoms including mood.

### Effects on stress and anxiety

Seven of the ten included studies assessed anxiety and stress, and all but two reported improvements after treatment with a probiotic. In addition to mood, Rao and colleagues, Chung and colleagues, and Messaoudi and colleagues also assessed stress and/or anxiety. As mentioned above, Rao and colleagues found that consumption of probiotics significantly improved anxiety, reflected in lower BAI scores [43]. However, when Chung and colleagues assessed stress in their older adult sample, there was no significant change in perceived stress scale (PSS) scores [44]. Similarly, Messaoudi and colleagues’ 2011 study also found no significant change on PSS scores after the introduction of their probiotic supplement, but secondary analyses showed that the reduced HSCL-90 and HADS scores held up in a subset of subjects with the lowest levels of stress, as measured by urinary free cortisol (UFC) levels [47]. Moreover, Gruenwald and colleagues’ 6-month study evaluating the effects of a probiotic multivitamin in 42 adults suffering from stress and exhaustion found that participants’ general condition improved by 41%, with a mean increase of 17% in positive condition and a mean decrease of 23% in negative condition [48]. Improvement in general condition of 22% was apparent by month 2 and reached 36% by month 4, with values for activity, elation, fatigue, and agitation contributing to these changes. 73% of participants rated the treatment’s effect as “good” or “very good” [36]. In 2004, Marcos and colleagues examined the effects of yogurt cultures with added probiotics on subjects under academic examination stress, and while numbers of lymphocytes and CD56 cells increased and decreased, respectively, levels of stress and anxiety, assessed using the state-trait anxiety inventory (STAI) remained unchanged [49]. Recently, Matthew Hilimire and colleagues took a different approach and surveyed 710 young adults using self-report questionnaires on fermented food consumption as well as neuroticism and social anxiety, using the big five inventory (BFI) and the social phobia and anxiety inventory (SPAI-23). The results showed that consumption of fermented foods containing probiotics was negatively associated with symptoms of social anxiety, and that consumption of fermented foods interacts with neuroticism to predict social anxiety symptoms. Those at higher genetic risk for social anxiety (indexed by high neuroticism) showed fewer social anxiety symptoms when they consumed more fermented foods [50].

### Effects on cognition

Three studies assessed some aspect of cognition, and all three of them reported positive effects. Messaoudi and colleagues assessed coping, defined as ‘the various cognitive or behavioral effects intended to master or tolerate the internal or external demands which threaten or go beyond the resources of a subject,’ with the coping checklist (CCL), and demonstrated that consumption of probiotics reduced self-blame scores and increased focus on problem-solving [36]. Chung and colleagues assessed cognition using the digit-span test (DST; to measure attention and working memory), story recall test (SRT), and verbal-learning test (VLT; to measure short- and long-term memory), as well as a rapid visual information-processing (RVIP) task and Stroop color-word test to measure cognitive fatigue. Results found a significant improvement on cognitive fatigue measures in those treated with the probiotic-fermented milk [44]. Interestingly, Benton and colleagues found no effect of the probiotic-containing milk drink on memory scores as measured with the Wechsler Memory Scale, but subjects who consumed the placebo had significantly better memory scores post-treatment. There was also no effect on verbal fluency [42].

## Discussion

Taken together, the findings from the included studies demonstrate that it is likely that daily consumption of a probiotic supplement could have a positive effect in improving the mood, anxiety, and cognitive symptoms present in MDD. The majority of the studies found positive results with no serious adverse events being reported. It appears that probiotics may have the most significant effect on symptoms of anxiety, which is often co-morbid with MDD [1]. This is consistent with research in animal models that report similar findings [26, 32, 35, 36], and in accordance with HPA activity in anxiety disorders. The sample characteristics from each study varied slightly. Seven studies assessed healthy controls, with one assessing MDD patients, one assessing CFS patients, and one assessing participants who were experiencing severe stress or exhaustion. All ten studies assessed adults, male and female, with some specifically focusing on young adults or older adults, but all between the age range of 18–75 years. Disparities in gender and age effects of probiotics are not well understood.

### Alternative treatment?

The use of probiotics as an alternative or adjuvant treatment for relieving symptoms of MDD and anxiety could be a critical turning point in the management of the disorder [23]. Because MDD is such a heterogeneous disorder, several problems exist with current antidepressant medications. While the physiological effects of most antidepressants, such as selective serotonin reuptake inhibitors (SSRIs), occur immediately after administration of the drug, the therapeutic effect can take weeks to become apparent in those seeking relief from symptoms. Even when they start to become effective, the side effects associated with them cause an estimated 15–30% of patients to discontinue their use [51]. Moreover, the perceived stigma associated with MDD and the use of antidepressants prevents individuals from seeking and adhering to treatment [52]. Several new terms have been introduced into the realm of mental health treatment and the gut–brain axis, which should not be confused with probiotics. Encephalobiotics, for example, is an umbrella term that encompasses probiotics, prebiotics, postbiotics, microbes, microbial parts, and/or agents that influence the microbiome for cognition, mental well-being, and brain health [53]. Psychobiotics on the other hand have been defined by Dinan and colleagues as live organisms that, when ingested in adequate amounts, produce a health benefit in patients suffering from psychiatric illness [54]. Psychobiotics would thus be classified as highly regulated drugs and may still harbor stigma the way an over-the-counter probiotic supplement may not.

Using probiotics to alleviate symptoms of MDD could indeed eliminate some of these barriers for effective treatment, though there is still much to be accomplished before probiotics can be considered a front-line treatment for alleviating depressive symptoms, including investigating the mechanisms underlying their effects.

### Proposed mechanisms of action

Throughout the literature, there appear to be two dominant hypotheses addressing possible mechanisms of action through which probiotics exert their effects on mood and cognition. These theories involve the regulation of inflammatory markers and the neurotransmission of the previously mentioned serotonin. However, as the immune system, central nervous system, and enteric nervous system are so intricately connected, it is possible that these two proposed mechanisms work in tandem to produce the effects induced by probiotics.

#### Inflammatory markers

Increased expression of pro-inflammatory cytokines IL-1β, IL-6, TNF-α, as well as interferon gamma (IFN-γ), and C-reactive protein (CRP) is repeatedly observed in patients suffering from depression [55–57] and has been associated with specific symptoms of depression [58, 59]. This overall increase in inflammation contributes to depressive symptoms by activating the HPA axis, as well as reducing the availability of neurotransmitter precursors and altering neurotransmitter metabolism. It is hypothesized that this inflammation is caused by increased intestinal permeability, or “leaky gut.” When the tight junctions of the gastrointestinal lining become compromised and permeability increases, it allows toxins and other forms of waste to leak into the bloodstream. Namely, gut-derived endotoxins called lipopolysaccharides (LPS)—molecules found in the outer membrane of gram-negative bacteria. These endotoxins trigger immune activation through Toll-like receptor 4 (TLR4) [60], causing the body to mount a global immune response. It is hypothesized that probiotics may exert their therapeutic effects on the central nervous system by improving the integrity of the gastrointestinal lining, reducing the ability of endotoxins to leak into the bloodstream and in turn, decreasing global inflammation. The reduction of this inflammation may result in improved regulation of the HPA axis and neurotransmitter activity.

#### Serotonin

Serotonin, or 5-hydroxytryptamine (5-HT), is a monoamine neurotransmitter implicated in the aetiology and pathophysiology of MDD [61] and is the main target of SSRIs. Serotonin is biosynthesized from the essential amino acid tryptophan, both in the central nervous system and the gastrointestinal tract. In the central nervous system, it is involved primarily in regulating stress and emotions, appetite, and sleep. In the gastrointestinal tract, it is responsible for key functions such as gastrointestinal motility and intestinal secretions. Alterations in the microbiome have been shown to profoundly influence neurotransmission of serotonin in both the peripheral and central nervous system. It is hypothesized that probiotics in the GI tract improve central nervous system symptoms associated with MDD by increasing production of free tryptophan, and in turn increasing serotonin availability. This increase in serotonin may facilitate regulation of the HPA axis and reduce depressive symptoms caused by a depletion of the neurotransmitter.

### Gaps in current research

Gaps and inconsistencies in the research on the effects of probiotics on depressive symptoms make it difficult to confirm evidence of efficacy. Duration of intervention varies widely, as does the quantity and strains of the probiotics, and research is lacking on depressive symptoms apart from mood, anxiety, and cognition, such as sleep. Perhaps most notably are the inconsistencies in defining depression across studies.

#### Strains

There are many different species and strains of probiotics that inhabit your gut and that are readily available in certain foods and in supplements alike, but the beneficial effects on health that probiotics confer are species- and strain-specific. Many *Lactobacillus* and *Bifidobacterium* strains have been studied in respect to mental health and seem to show the most beneficial effects [62], as opposed to other species such as *Streptococcus* and *Bacillus*, but we have not yet identified the most efficacious strains or combinations of strains for improving mental health. Both *L. helveticus* and *B. longum* have been shown to affect the gut–brain axis [63], but several studies exploring other strains of *Lactobacillus* and *Bifidobacterium* and garner similar results. However, many of these studies use a combination of strains, making it impossible to isolate which strain or strains may be exerting the effects. Further research in the form of blinded and controlled studies assessing individual strains is required to identify strains that may have positive effects on mental health.

#### Dosing

 Probiotic dosing is based on the number of live organisms, referred to as colony forming units (CFU). Dosage may be species- and strain-dependent, with some species such as *Bifidobacterium infantis* 35,624 requiring 10^8^ CFU to produce beneficial effects to treat irritable bowel syndrome [64], but research is lacking on effective dosages for other species. It is also important to note that most prescribed doses are based on the treatment of GI disorders, and given that dosing may depend on the indication the probiotics are being prescribed for, they could differ for effective relief of depressive symptoms. After determining the most efficacious strains for mental health purposes, we then need to assess the strains in different doses to determine the ideal amount of CFU.

#### Duration of intervention

The duration of intervention with probiotics varies widely. While typical antidepressant clinical trials last approximately 6 weeks [65], the ideal duration to see specific effects of probiotics is unknown. As with the strain and dosing, it is possible that duration of treatment is also dependent on indication.

#### Sleep

Impaired sleep is a poignant symptom of MDD, most commonly manifested in difficulty falling asleep, difficulty staying asleep, unrefreshing sleep, and daytime sleepiness [66]. Serotonin, which is dysregulated in MDD, is a neuromodulator of sleep. Considering probiotics in the GI tract have been shown to affect levels of serotonin via the gut–brain axis, and changes in intestinal microbiota have been shown to improve sleep parameters in CFS patients [67], it seems likely that probiotics could have an effect on sleep as well, yet this has not been explored.

#### Defining depression

Depression is an umbrella term for several different disorders, including MDD, bipolar disorder, seasonal affective disorder, dysthymia, and postpartum depression. These disorders are not simply a passing low mood, but significantly interfere in daily life and normal functioning, and although they do have overlapping symptoms, are each unique disorders. In most of the studies, the term ‘depression’ is poorly defined or not clarified. It is important that the type of depression is clearly defined so that it can be differentiated from other types of depressive disorders as well as sub-clinical depressive symptoms. Research on sub-clinical depression is equally important, as low-grade depressive symptoms also significantly affect performance at work [68], cause household, social, and financial strain [69], and are associated with decreased quality of life [70] and suicidal ideation [71]. These subsyndromal depressions are often ignored in research or not defined adequately.

## Conclusions and future directions

The robust evidence compiled and presented in this review indicates that treatment with probiotics may improve symptoms associated with MDD by increasing serotonin availability and/or decreasing levels of inflammatory markers. The potential of probiotics to be used as a novel treatment for MDD could have a major impact on those seeking antidepressant treatment by reducing the stigma, latency and side effects associated with typical antidepressants. Despite extensive preclinical data, the clinical effects of probiotics on mental health have yet to be studied comprehensively in a sample of depressed patients. Further research is warranted to determine probiotics’ efficacy for alleviating depressive symptoms, as well as the ideal duration of treatment, dosage, and strain of probiotic for achieving efficacy in terms of mental health. In addition, the effect of probiotics on sleep should be explored and the term depression should be clearly defined and diagnosed. Additional double-blind randomized controlled trials in clinical psychiatric samples are required in order to shed more light on this topic.

## Abbreviations

ACTH: adrenocorticotrophic hormone
BAI: Beck Anxiety Inventory
BDI: Beck Depression Inventory
BDNF: brain-derived neurotrophic factor
BFI: big five inventory
CCL: coping checklist
CFS: chronic fatigue syndrome
CFU: colony forming unit
CINAHL: cumulative index to nursing and allied health literature
CRP: C-reactive protein
DST: digit span test
GABA: gamma-aminobutyric acid
GDS-SF: geriatric depression scale
GI: gastrointestinal
HADS: Hospital Anxiety and Depression Scale
HPA: hypothalamic–pituitary adrenal (axis)
HSCL-90: Hopkins symptom checklist
IFN- γ: interferon-gamma
IL-1β: interleukin-1-beta
IL-6: interleukin-6
LEIDS-R: Leiden index of depression sensitivity
LPS: lipopolysaccharide
MDD: major depressive disorder
POMS: profile of mood states
PRISMA: preferred reporting items for systematic reviews and meta-analyses
PSS: perceived stress scale
RVIP: rapid visual information-processing
SPAI-23: social phobia and anxiety inventory
SRT: story recall test
SSRI: selective serotonin reuptake inhibitor
STAI: state-trait anxiety inventory
TLR4: toll-like receptor 4
TNFα: tumor necrosis factor-alpha
UFC: urinary free cortisol
VLT: verbal learning test
WoS: web of science
5-HIAA: 5-hydroxyindoleacetic acid
5-HT: 5-hydroxytryptamine

## References

[1] Kessler RC, Chiu WT, Demler O (2005). Prevalence, severity, and comorbidity of 12-month DSM-IV disorders in the National Comorbidity Survey Replication. Arch Gen Psychiatry.

[2] Kessler RC, Bromet EJ (2013). The epidemiology of depression across cultures. Annu Rev Public Health.

[3] Anderson HD, Pace WD, Libby AM (2012). Rates of 5 common antidepressant side effects among new adult and adolescent cases of depression: a retrospective US claims study. Clin Ther.

[4] Foster JA, Neufeld KAM (2013). Gut–brain axis: how the microbiome influences anxiety and depression. Trends Neurosci.

[5] Jiang H, Ling Z, Zhang Y (2015). Altered fecal microbiota composition in patients with major depressive disorder. Brain Behav Immun.

[6] Naseribafrouei A, Hestad K, Avershina E (2014). Correlation between the human fecal microbiota and depression. Neurogastroenterol Motil.

[7] Kelly JR, Borre Y, O’Brien C (2016). Transferring the blues: depression-associated gut microbiota induces neurobehavioural changes in the rat. J Psychiatr Res.

[8] Walker EA, Katon WJ, Jemelka RP (1992). Comorbidity of gastrointestinal complaints, depression, and anxiety in the epidemiologic catchment area (ECA) study. Am J Med.

[9] Mangiola F, Ianiro G, Franceschi F (2016). Gut microbiota in autism and mood disorders. World J Gastroenterol.

[10] Goodrich JK, Waters JL, Poole AC (2014). Human genetics shape the gut microbiome. Cell.

[11] Yatsunenko T, Rey FE, Manary MJ (2012). Human gut microbiome viewed across age and geography. Nature.

[12] Markle JG, Frank DN, Mortin-Toth S (2013). Sex differences in the gut microbiome drive hormone-dependent regulation of autoimmunity. Science.

[13] David LA, Maurice CF, Carmody RN (2014). Diet rapidly and reproducibly alters the human gut microbiome. Nature.

[14] O’Mahony SM, Marchesi JR, Scully P (2009). Early life stress alters behavior, immunity, and microbiota in rats: implications for irritable bowel syndrome and psychiatric illnesses. Biol Psychiatry.

[15] Meddings JB, Swain MG (2000). Environmental stress-induced gastrointestinal permeability is mediated by endogenous glucocorticoids in the rat. Gastroenterology.

[16] Rhee SH, Pothoulakis C, Mayer EA (2009). Principles and clinical implications of the brain–gut–enteric microbiota axis. Nat Rev Gastroenterol Hepatol.

[17] Logan AC, Jacka FN (2014). Nutritional psychiatry research: and emerging discipline and its intersection with global urbanization, environmental challenges, and the evolutionary mismatch. J Physiol Anthropol.

[18] Selhub EM, Logan AC, Bested AC (2014). Fermented foods, microbiota, and mental health: ancient practice meets nutritional psychiatry. J Physiol Anthropol.

[19] Sanchez-Villegas A, Delgado-Rodriguez M, Alonso A (2009). Association of the Mediterranean dietary pattern with the incidence of depression: the Seguimiento Universidad de Navarra/University of Navarra follow-up (SUN) cohort. Arch Gen Psychiatry.

[20] Akbaraly TN, Brunner EJ, Ferrie JE (2009). Dietary pattern and depressive symptoms in middle age. Br J Psychiatry.

[21] Dinan TG, Quigley EM (2011). Probiotics in the treatment of depression: science or science fiction?. Aust N Z J Psychiatry.

[22] Parvez S, Malik KA, Ah Kang S (2006). Probiotics and their fermented food products are beneficial for health. J Appl Microbiol.

[23] Logan AC, Katzman M (2005). Major depressive disorder: probiotics may be an adjuvant therapy. Med Hypotheses.

[24] Ait-Belgnaoui A, Durand H, Cartier C (2012). Prevention of gut leakiness by a probiotic treatment leads to attenuated HPA response to an acute psychological stress in rats. Psychoneuroendocrinology.

[25] Ait-Belgnaoui A, Colom A, Braniste V (2014). Probiotic gut effect prevents the chronic psychological stress-induced brain activity abnormality in mice. Neurogastroenterol Motil.

[26] Bravo JA, Forsythe P, Chew MV (2011). Ingestion of Lactobacillus strain regulates emotional behavior and central GABA receptor expression in a mouse via the vagus nerve. PNAS.

[27] Stetler C, Miller GE (2011). Depression and hypothalamic–pituitary–adrenal activation: a quantitative summary of four decades of research. Psychosom Med.

[28] Sherwin E, Rea K, Dinan TG (2016). A gut (microbiome) feeling about the brain. Curr Opin Gastroenterol.

[29] Sen S, Duman R, Sanacora G (2008). Serum brain-derived neurotrophic factor, depression, and antidepressant medications: meta-analyses and implications. Biol Psychiatry.

[30] Desbonnet L, Garrett L, Clarke G (2008). The probiotic *Bifidobacteria infantis*: an assessment of potential antidepressant properties in the rat. J Psychiatr Res.

[31] Nishino R, Mikami K, Takahashi H (2013). Commensal microbiota modulate murine behaviors in a strictly contamination-free environment confirmed by culture-based methods. Neurogastroenterol Motil.

[32] Luo J, Wang T, Liang S (2014). Ingestion of Lactobacillus strain reduces anxiety and improves cognitive function in the hyperammonemia rat. Sci China Life Sci.

[33] Savignac HM, Tramullas M, Kiely B (2015). Bifidobacteria modulate cognitive processes in an anxious mouse strain. Behav Brain Res.

[34] Smith CJ, Emge JR, Berzins K (2014). Probiotics normalize the gut–brain–microbiota axis in immunodeficient mice. Am J Physiol Gastrointest Liver Physiol.

[35] Kantak PA, Bobrow DN, Nyby JG (2014). Obsessive-compulsive-like behaviors in house mice are attenuated by a probiotic (*Lactobacillus rhamnosus* GG). Behav Pharmacol.

[36] Messaoudi M, Lalonde R, Violle N (2011). Assessment of psychotropic-like properties of a probiotic formulation (*Lactobacillus helveticus* R0052 and *Bifidobacterium longum* R0175) in rats and human subjects. Br J Nutr.

[37] Li S, Zhao Y, Zhang Y (2012). Antioxidant activity of *Lactobacillus plantarum* strains isolated from traditional Chinese fermented foods. Food Chem.

[38] Dhakal R, Bajpai VK, Baek KH (2012). Production of GABA by microorganisms: a review. Braz J Microbiol.

[39] Jumpertz R, Le DS, Turnbaugh PJ (2011). Energy-balance studies reveal associations between gut microbes, caloric load, and nutrient absorption in humans. Am J Clin Nutr.

[40] Cepeda MS, Katz EG, Blacketer C. Microbiome–gut–brain axis: probiotics and their association with depression. J Neuropsychiatry Clin Neurosci 2016; appi-neuropsych. 10.1176/appi.neuropsych.15120410 27539375

[41] Jadad AR, Moore RA, Carroll D (1996). Assessing the quality of reports of randomized clinical trials: is blinding necessary?. Control Clin Trials.

[42] Benton D, Williams C, Brown A (2007). Impact of consuming a milk drink containing a probiotic on mood and cognition. Eur J Clin Nutr.

[43] Rao AV, Bested AC, Beaulne TM (2009). A randomized, double-blind, placebo-controlled pilot study of a probiotic in emotional symptoms of chronic fatigue syndrome. Gut Pathogens.

[44] Chung YC, Jin HM, Cui Y (2014). Fermented milk of *Lactobacillus helveticus* IDCC3801 improves cognitive functioning during cognitive fatigue tests in healthy older adults. J Funct Foods.

[45] Steenbergen L, Sellaro R, van Hemert S (2015). A randomized controlled trial to test the effect of multispecies probiotics on cognitive reactivity to sad mood. Brain Behav Immun.

[46] Akkasheh G, Kashani-Poor Z, Tajabadi-Ebrahimi M (2016). Clinical and metabolic response to probiotic administration in patients with major depressive disorder: a randomized, double-blind, placebo-controlled trial. Nutrition.

[47] Messaoudi M, Violle N, Bisson JF (2011). Beneficial psychological effects of a probiotic formulation (*Lactobacillus helveticus* R0052 and *Bifidobacterium longum* R0175) in healthy human volunteers. Gut Microbes.

[48] Gruenwald J, Graubaum HJ, Harde A (2002). Effect of a probiotic multivitamin compound on stress and exhaustion. Adv Ther.

[49] Marcos A, Wärnberg J, Nova E (2004). The effect of milk fermented by yogurt cultures plus *Lactobacillus casei* DN-114001 on the immune response of subjects under academic examination stress. Eur J Nutr.

[50] Hilimire MR, DeVylder JE, Forestell CA (2015). Fermented foods, neuroticism, and social anxiety: an interaction model. Psychiatry Res.

[51] Gartlehner G, Hansen RA, Carey TS (2005). Discontinuation rates for selective serotonin reuptake inhibitors and other second-generation antidepressants in outpatients with major depressive disorder: a systematic review and meta-analysis. Int Clin Psychopharmacol.

[52] Sirey JA, Bruce ML, Alexopoulos GS (2001). Stigma as a barrier to recovery: perceived stigma and patient-rated severity of illness as predictors of antidepressant drug adherence. Psychiatr Serv.

[53] Prescott SL, Logan AC (2016). Transforming life: a broad view of the developmental origins of health and disease concept from an ecological justice perspective. Int J Environ Res Public Health.

[54] Dinan TG, Stanton C, Cryan JF (2013). Psychobiotics: a novel class of psychotropic. Biol Psychiatry.

[55] Maes M, Scharpé S, Meltzer HY (1994). Increased neopterin and interferon-gamma secretion and lower availability of l-tryptophan in major depression: further evidence for an immune response. Psychiatry Res.

[56] Owen BM, Eccleston D, Ferrier IN (2001). Raised levels of plasma interleukin-1β in major and postviral depression. Acta Psychiatr Scand.

[57] Howren MB, Lamkin DM, Suls J (2009). Associations of depression with C-reactive protein, IL-1, and IL-6: a meta-analysis. Psychosom Med.

[58] Anisman H, Ravindran A, Griffiths J (1999). Endocrine and cytokine correlates of major depression and dysthymia with typical or atypical. Mol Psychiatry.

[59] Yirmiya R (1997). Behavioral and psychological effects of immune activation: implications for ‘depression due to a general medical condition’. Curr Opin Psychiatry.

[60] Kawai T, Takeuchi O, Fujita T (2001). Lipopolysaccharide stimulates the MyD88-independent pathway and results in activation of IFN-regulatory factor 3 and the expression of a subset of lipopolysaccharide-inducible genes. J Immunol.

[61] Owens MJ, Nemeroff CB (1994). Role of serotonin in the pathophysiology of depression: focus on the serotonin transporter. Clin Chem.

[62] Mayer EA, Knight R, Mazmanian SK (2014). Gut microbes and the brain: paradigm shift in neuroscience. J Neurosci.

[63] Diop L, Guillou S, Durand H (2008). Probiotic food supplement reduces stress-induced gastrointestinal symptoms in volunteers: a double-blind, placebo-controlled, randomized trial. Nutr Res.

[64] Whorwell PJ, Altringer L, Morel J (2006). Efficacy of an encapsulated probiotic *Bifidobacterium infantis* 35624 in women with irritable bowel syndrome. Am J Gastreoenterol.

[65] Quitkin FM, Rabkin JG, Ross D (1984). Duration of antidepressant drug treatment: what is an adequate trial?. Arch Gen Psychiatry.

[66] Paterson LM, Nutt DJ, Wilson SJ (2009). NAPSAQ-1: National Patient Sleep Assessment Questionnaire in depression. Int J Psychiatry Clin Pract.

[67] Jackson ML, Butt H, Ball M (2015). Sleep quality and the treatment of intestinal microbiota imbalance in chronic fatigue syndrome: a pilot study. Sleep Science.

[68] Martin JK, Blum TC (1996). Subclinical depression and performance at work. Soc Psychiatry Psychiatr Epidemiol.

[69] Judd LL, Paulus MP, Wells KB (1996). Socioeconomic burden of subsyndromal depressive symptoms and major depression in a sample of the general population. Am J Psychiatry.

[70] Chachamovich E, Fleck M, Laidlaw K (2008). Impact of major depression and subsyndromal symptoms on quality of life and attitudes toward aging in an international sample of older adults. Gerontologist.

[71] Cukrowicz KC, Schlegel EF, Smith PN (2011). Suicide ideation among college students evidencing subclinical depression. J Am Coll Health.

