# Erratum to: The effects of probiotics on depressive symptoms in humans: a systematic review

**DOI:** 10.1186/s12991-017-0141-7

**Published:** 2017-03-07

**Authors:** Caroline J. K. Wallace, Roumen Milev

**Affiliations:** Department of Psychiatry, Queen’s University, 752 King Street West, Kingston, ON K7L 4X3 Canada

## Erratum to: Ann Gen Psychiatry (2017) 16:14 DOI 10.1186/s12991-017-0138-2

This article [1] has been updated to include the correct version of Table 1. The article originally published online showed an incomplete version of the table. The correct version of Table 1 is shown in this erratum. This error was carried forward by the production team and was not the fault of any authors.

**Table 1:** Characteristics of included studies

Reference	Sample characteristics	Strain	Study design	Duration of intervention	Measurement	Key findings and conclusions
Akkasheh et al. [34]	40 MDD patients. Age 20–55 years	*Lactobacillus acidophilus*,* L. casei*, and* Bifidobacterium bifidum*	Double-blind, randomized, placebo-controlled trial	8 weeks	BDI	Consumption of probiotic supplement improved BDI scores
Benton et al. [30]	124 healthy humans. Avg. age 62 years	*L. casei*	Double-blind, randomized, placebo-controlled trial	3 weeks	POMS, self-rated mood	No effect of probiotic on POMS results. Consumption of probiotic-containing yogurt improved self-reported mood of those whose mood was initially poor
Chung et al. [32]	36 healthy humans. Age 60–75 years	*L. helveticus*	Double-blind, randomized, placebo-controlled trial	12 weeks	PSS, GDS-SF, DST, SRT, VLT, RVIP, Stroop Task	No significant effects of probiotics on the PSS, GDS-SF. Consumption of probiotics did improve DST, SRT, VLT, RVIP, and Stroop Tasks scores
Gruenwal et al. [36]	34 adults suffering from stress or exhaustion. Mean age 44 years	*L. acidophilus* and *B. bifidum* and *longum*	Pre- and post-intervention assessment	6 months	PNQ, EWL	Subjects’ general condition improved by 40.7%. 73% of participants rated the effect of treatment as “good” or “very good”
Hilimire et al. [38]	710 young adults. Mean age 19 years	Unknown	Self-report questionnaires on fermented food consumption, neuroticism and social anxiety	N/A	BFI, SPAI-23	Consumption of fermented foods containing probiotics was negatively associated with symptoms of social anxiety and interacts with neuroticism to predict social anxiety symptoms. Those at higher genetic risk for social anxiety disorder (indexed by high neuroticism) show fewer social anxiety symptoms when they consume more fermented foods
Marcos et al. [37]	136 healthy students. Age 18–23 years	*L. casei*	Prospective, randomized, controlled, parallel study	6 weeks	STAI	No significant effects of probiotics on anxiety levels. Probiotics did modulate lymphocyte and CD56 cell counts
Messaoudi et al. [28]	55 healthy Caucasians. Mean age 43 years	*L. helveticus* and *B. longum*	Double-blind, randomized, controlled, parallel study	30 days	HADS, HSCL-90, PSS, CCL	Consumption of probiotics reduced global severity index of the HSCL-90 due to lower somatization, depression, and anger-hostility and also reduced HADS global scores. Consumption of probiotic reduced self-blame score on CCL and increased focus on problem solving. No effect on PSS
Messaoudi et al. [35]	Sub-population of above sample of 25 with lowest UFC levels	*L. helveticus* and *B. longum*	Double-blind, randomized, controlled, parallel study	30 days	HADS, HSCL-90	Consumption of probiotics reduced HADS and HSCL-90 scores
Rao et al. [31]	35 CFS patients. Age 18–65 years	*L. casei*	Double-blind, randomized, placebo-controlled pilot study	2 months	BDI, BAI	Consumption of probiotics significantly improved BAI scores. No effect on BDI scores
Steenbergen et al. [33]	40 non-smoking healthy young adults. Mean age 20 years	*B. lactis* and *L. acidophilus*,* brevis*,* casei*,* lactis*, and *salivarius*	Triple-blind, randomized, placebo-controlled, pre- and post-intervention assessment	4 weeks	LEIDS-r	Consumption of multispecies probiotic significantly reduced overall cognitive reactivity to depression (in particular aggressive and ruminative thoughts)

*MDD* major depressive disorder, *BDI* Beck Depression Inventory, *POMS* profile of mood states scale, *PSS* perceived stress scale, *GDS*-*SF* geriatric depression scale, *DST* digit span test, *SRT* story recall test, *VLT* verbal learning test, *RVIP* rapid visual information-processing, *PNQ* psychological-neurologic questionnaire, *EWL* list of adjectives, *BFI* big five inventory, *SPAI-23* social phobia and anxiety inventory, *STAI* state-trait anxiety inventory, *HADS* Hospital Anxiety Depression Scale, *HSCL-90* Hopkins symptom checklist, *CCL* coping checklist, *UFC* urinary free cortisol, *BAI* Beck Anxiety Inventory, *LEIDS*-*r* Leiden index of depression sensitivity, *fMRI* functional magnetic resonance imaging
